# Spontaneous Septostomy in a Twin Pregnancy Causing Fatal Amniotic Band Syndrome

**DOI:** 10.1155/2018/4549060

**Published:** 2018-12-30

**Authors:** Carolina Hvelplund, Kasper Pihl, Simon Trautner, Pernille Pedersen, Lisa Leth Maroun

**Affiliations:** ^1^Neonatal Intensive Care Unit, Department of Pediatrics, Copenhagen University Hospital Hvidovre, Hvidovre, Denmark; ^2^Department of Gynecology and Obstetrics, Copenhagen University Hospital Hvidovre, Hvidovre, Denmark; ^3^Neonatal Department, Juliane Marie Centre, Copenhagen University Hospital Rigshospitalet, Copenhagen, Denmark; ^4^Department of Pathology, Copenhagen University Hospital Rigshospitalet, Copenhagen, Denmark

## Abstract

Complications due to spontaneous septostomy of the dividing membrane in monochorionic diamniotic pregnancies are rarely described. Herein, we report the case of a preterm female neonate from a monochorionic diamniotic twin pregnancy delivered by caesarean section at 32 weeks of gestation. She was born with a broad band of a transparent membrane-like material firmly attached to her lower abdomen. Postnatally, she developed respiratory distress syndrome and persistent pulmonary hypertension, complicated by bilateral pneumothorax. She died due to respiratory failure when she was 1 day old. Her twin sister survived with no malformations. At postmortem examination, the neonate had severe lung hypoplasia, and the attached material was diagnosed as the dividing septum. We hypothesize that the lung hypoplasia was secondary to local oligohydramnios, which developed as a consequence of the twin being firmly stuck in the defect of the dividing membrane. To our best knowledge, spontaneous septostomy causing an ultimately fatal amniotic band syndrome has not previously been described.

## 1. Introduction

Spontaneous rupture (septostomy) of the dividing membrane in twin pregnancies is a rare event. Few cases have been reported, predominantly in monochorionic diamniotic (MCDA) twin pregnancies [1, 2]. Antenatal diagnosis of a septostomy remains a challenge and requires a high index of suspicion during an ultrasonography. Septostomy is typically identified postnatally because of entangled umbilical cords [3]. Amniotic band syndrome (ABS) is a rare condition where fetal parts, typically the limbs, become entangled by amniotic bands that may cause deformation, constriction, and even amputation. The association between spontaneous septostomy and ABS has not been described. Herein, we report a case of an MCDA twin pregnancy complicated by spontaneous septostomy causing fatal ABS in one twin.

## 2. Case Presentation

A 38-year-old nulliparous woman was pregnant with MCDA twins. The pregnancy was conceived after in vitro fertilization. Ultrasonography (US) was performed at 20 weeks (normal malformation scan) and again at 23 weeks showing intrauterine growth restriction of both twins. A normal fetal echocardiography was performed at week 28. At 31 weeks, the fetal US still showed intrauterine growth restriction (twin A 26% and twin B 31%). It also revealed mild dilation of the gut in twin A.

**Figure 1:**
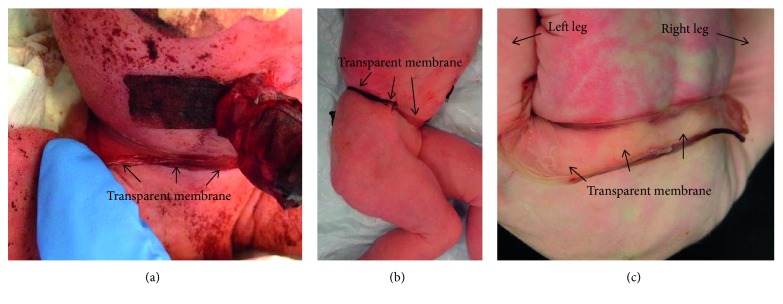
(a–c) The broad band of a transparent membrane-like material firmly attached to twin A's lower truncus. (a) Ventral view after birth. (b) Transparent membrane. (c) Right side view on autopsy.

The mother developed severe preeclampsia around week 28 and was hospitalized. Antenatal corticosteroids were administered to aid fetal lung maturation. Her preeclampsia progressed, and the mother developed hemolysis, elevated liver enzymes, and low platelet count (HELLP syndrome). Caesarean section was performed at a gestational age of 32 weeks and 0 days because of HELLP syndrome. Two live female neonates were delivered. Both twins were small for gestational age with a birth weight of 1200 g for twin A (−3, 25 standard deviation (SD)) [4] and 1290 g for twin B (−2, 85 SD). Twin A showed a broad band of a transparent membrane-like material firmly attached to her lower abdomen (Figure 1(a)). She had normal movement and circulation of the lower limbs. Her twin sister showed no malformations and is well after follow-up at 7 months of chronological age.

Twin A developed respiratory distress syndrome. Initially, she was stable and treated with nasal continuous positive airway pressure. She subsequently developed apneic episodes and increased oxygen requirement and was treated with surfactant. She was mechanically ventilated and developed persistent pulmonary hypertension, complicated by bilateral pneumothorax, which was treated with bilateral needle aspiration and unilateral chest tube. Umbilical lines were inserted, and an isotonic crystalloid solution was given as volume expander. The blood glucose level was normal. Antibiotics were administered. Despite the maximal treatment, she developed hypoxia and increasing metabolic acidosis. Because of twin A's high ventilatory requirement, both infants were transferred by the neonatal transport service to a tertiary neonatal intensive care unit for further stabilization and treatment.

At the tertiary neonatal intensive care unit, treatment of twin A with nitric oxide was initiated, a second chest tube was inserted, and inotropic treatment combined with systemic steroids was established, all without significant effects. She developed increasing lactic acidosis despite bilateral chest tubes, inotropic support, nitric oxide, and volume therapy. With the parents' agreement, intensive support was withdrawn, and twin A died on her second day of life. The family granted permission for postmortem examination.

**Figure 2:**
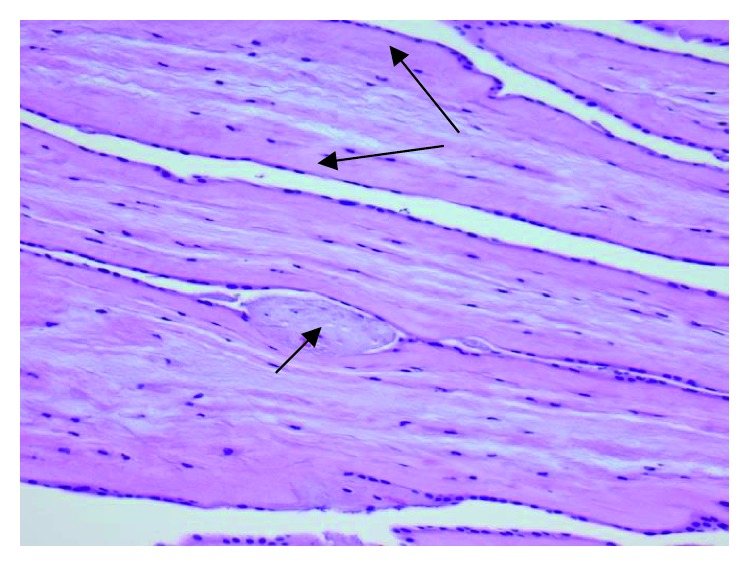
Histopathological examination of a roll of the transparent membrane showed double amnion layer (long arrows) without chorion, consistent with monochorionic diamniotic pregnancy. On one side, there was amnion nodosum (short arrow) consistent with oligohydramnios.

On autopsy, twin A showed signs of intrauterine growth restriction with body dimensions appropriate for 30–32 weeks of gestation. A broad band of a transparent membrane was found firmly attached in a circumferential severe stricture on the lower trunk at the level of the back, hips, and lower abdomen (Figures 1(b) and 1(c)). No other strictures or amniotic bands were seen. There was pneumothorax, the lungs were hypoplastic, and their weights corresponded to 24 weeks of gestation. Microscopy of the lung tissue showed severe hyaline membrane disease. The attached band-like material was identified as the dividing membrane of the MCDA pregnancy, on one side displaying amnion nodosum as a sign of oligohydramnios (Figure 2). The placenta was not available for examination.

## 3. Discussion

The postmortem findings and clinical information in this unusual case suggest that the lower body of twin A must have passed through a spontaneous rupture of the dividing membrane into the amniotic cavity of twin B. Our hypothesis is that twin A was firmly stuck in the defect for some time. Aspiration of fluid from the gestational sac of twin A combined with urination into the sac of twin B most probably led to local oligohydramnios around the head and thorax of twin A, thereby causing lung hypoplasia. Our case represents a rare form of ultimately fatal amniotic band syndrome, resulting from antepartum rupture of the dividing membrane between the twins.

In the Danish population, approximately 1% of all naturally conceived pregnancies result in twins [5]. More than 10% of perinatal deaths occur after pregnancies with multiple gestations, with prematurity and iatrogenic factors as the major causes. The highest mortality rate (30–70%) occurs in monochorionic monoamniotic pregnancies (1% of all twin pregnancies) due to the added risk of cord entanglement [6].

MCDA and dichorionic diamniotic (DCDA) twins are not usually thought to be at risk for cord entanglement; however, this may be so in the rare event of a spontaneous or iatrogenic rupture of the dividing membrane. The incidence of spontaneous septostomy in uncomplicated MCDA twin pregnancies is not known [7] but has been reported as high as 1.8% by one high-risk unit [6]. The causes of poor outcomes in twin pregnancies with intrauterine disruption of the dividing membrane have been described as including perinatal death secondary to preterm rupture of the membranes, prematurity, and cord entanglement [8]. A literature review of 15 reported cases of spontaneous septostomy by Fleming and Miller [2] included two cases, in which both twins died, and three cases, in which one twin survived and the other twin died or suffered significant hypoxic injury. The outcome was better for the nine cases where the septostomy was diagnosed antenatally by ultrasonography. Gilbert et al. [8] reported one case of ABS in one twin from a DCDA pregnancy with an intact septum, but we did not find reports of ABS associated with spontaneous septostomy.

The etiology of the antepartum rupture of the dividing membrane in our case is not clear. There were no history of infection or trauma, no sonographic signs of fetal disruption of the septum, and no amniocentesis or other intrauterine procedures to suggest pseudoamniotic band syndrome (PABS), which is an iatrogenic septostomy that occurs after invasive procedures in monochorionic twin pregnancies. The incidence of PABS is around 2% [5]. Sonographic signs of septostomy include twins occupying the same side of the dividing membrane and in twin-twin transfusion syndrome, polyhydramnios in the donor's sac despite a collapsed donor bladder and umbilical cord entanglement.

Our case is an example of a very unusual sequence of events leading to neonatal death in a case of spontaneous septostomy in MCDA twins. Our case illustrates the possible severe consequences of spontaneous rupture of the septum in twin pregnancies. To our knowledge, spontaneous septostomy causing a fatal ABS has not been reported before. Awareness of this complication might increase the likelihood of antenatal detection, potentially improving the outcome. Perinatal sonographers are advised to thoroughly look for signs of spontaneous septostomy during regular follow-up US for MCDA or DCDA twins.
